# Spatially offset Raman spectroscopy for biomedical applications†

**DOI:** 10.1039/d0cs00855a

**Published:** 2020-11-10

**Authors:** Fay Nicolson, Moritz F. Kircher, Nick Stone, Pavel Matousek

**Affiliations:** Department of Imaging, Dana-Farber Cancer Institute & Harvard Medical School Boston MA 02215 USA; Department of Radiology, Brigham & Women's Hospital & Harvard Medical School Boston MA 022115 USA; School of Physics and Astronomy, University of Exeter Exeter EX4 4QL UK n.stone@exeter.ac.uk; Royal Devon and Exeter NHS Foundation Trust Barrack Road Exeter Devon EX2 5DW UK; Central Laser Facility, Research Complex at Harwell, STFC Rutherford Appleton Laboratory, UKRI Harwell Oxford OX11 0QX UK pavel.matousek@stfc.ac.uk

## Abstract

In recent years, Raman spectroscopy has undergone major advancements in its ability to probe deeply through turbid media such as biological tissues. This progress has been facilitated by the advent of a range of specialist techniques based around spatially offset Raman spectroscopy (SORS) to enable non-invasive probing of living tissue through depths of up to 5 cm. This represents an improvement in depth penetration of up to two orders of magnitude compared to what can be achieved with conventional Raman methods. In combination with the inherently high molecular specificity of Raman spectroscopy, this has therefore opened up entirely new prospects for a range of new analytical applications across multiple fields including medical diagnosis and disease monitoring. This article discusses SORS and related variants of deep Raman spectroscopy such as transmission Raman spectroscopy (TRS), micro-SORS and surface enhanced spatially offset Raman spectroscopy (SESORS), and reviews the progress made in this field during the past 5 years including advances in non-invasive cancer diagnosis, monitoring of neurotransmitters, and assessment of bone disease.

## Abstract 2

Recent advances in non-invasive biomedical analysis using SORS are discussed.

## Graphical abstract

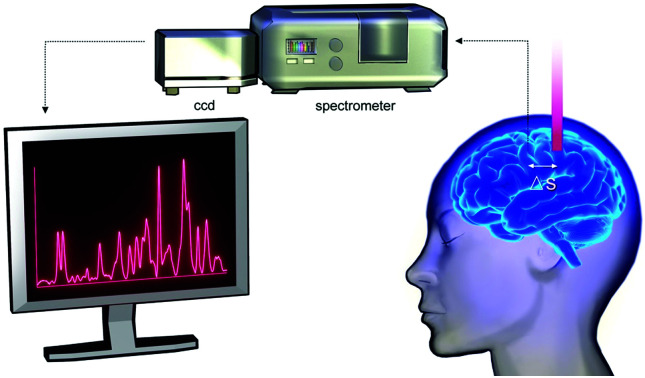

## Introduction

A number of medical conditions such as cancer and bone disorders are accompanied by significant chemical alterations in the composition of associated cells and tissues. These changes, however, facilitate their ability to be detected using chemically sensitive and specific analytical methods such as Raman spectroscopy. For the widest and easiest applicability, these approaches should be non-invasive, provide adequate sensitivity despite rapid acquisition, possess high accuracy, and be accessible at moderate costs.^1^ Currently, the most widely applied imaging approaches utilized in the clinic are computed tomography (CT), magnetic resonance imaging (MRI), positron emission tomography (PET), and ultrasound. Although these established imaging modalities are well suited to a range of medical applications, they are associated with at least one of the following limitations: high cost, *e.g.* MRI and PET; involve the use of ionising radiation, *e.g.* CT and PET; or provide inadequate molecularly specific information, *e.g.* CT, MRI and ultrasound.^1,2^

In this context Raman spectroscopy can offer high molecular specificity, rapid application as well as low to moderate cost. In addition, Raman spectroscopy avoids the safety concerns associated with ionising radiation, thus providing attractive, complementary alternatives to traditional imaging modalities. However, traditional Raman imaging is limited in its ability to probe deeper seated tissue at depth beyond near-surface tissue layers (typically several hundred micrometres deep to mm with “surface enhanced Raman scattering” (SERS) agents). Subcutaneous Raman needle probes have been developed for this purpose,^3,4^ but of course can only be employed invasively in non-sensitive tissues. Many clinical applications are more suited to non-invasive approaches, thus preventing Raman spectroscopy from being applied to diagnostic applications which require deeper scanning capabilities. The advent of “Spatially Offset Raman Spectroscopy” (SORS) and the development of its variants,^5,6^ now permits Raman imaging at up to an order of two magnitudes deeper than previously possible using conventional Raman techniques. SORS therefore enables tissue monitoring through depths of mm and cm non-invasively.^6^ This article reviews this rapidly expanding field from the perspective of biomedical diagnosis, specifically focusing on progress over the recent years and the techniques most relevant to the field of medical diagnostics.

## Deep-tissue Raman spectroscopy techniques

SORS and its related concepts have built upon preceding research and advances in NIR and fluorescence photon diffusion, which was dedicated to investigating photon propagation mechanisms in turbid media and developing deep probing approaches for these techniques.^7–13^ By utilising the inelastically scattered Raman photons generated as the laser photons travel through cells and tissues, it is possible to achieve much higher chemical specificity, broadly on a par with mid-infrared absorption spectroscopy, which itself cannot be used for deep probing of living tissue due to the high level of absorption of its signals by water and other components in tissues. Early research into the use of Raman spectroscopy for deep probing in turbid media included time-gated approaches which utilise the fact that in comparison to shallow born Raman photons, deeper born Raman photons take longer to emerge at the surface of a turbid medium.^14^ These, however, rely on the use of impulsive Raman excitation and time-gated detection, which is therefore instrumentally complex and costly. Moreover, the approach provides relatively moderate gains in depth penetration and is associated with significant safety issues due to the impulsive nature of the laser excitation which translate into restrictions to applicable powers in *in vivo* applications.

### Spatially offset Raman spectroscopy (SORS)

**Fig. 1:**
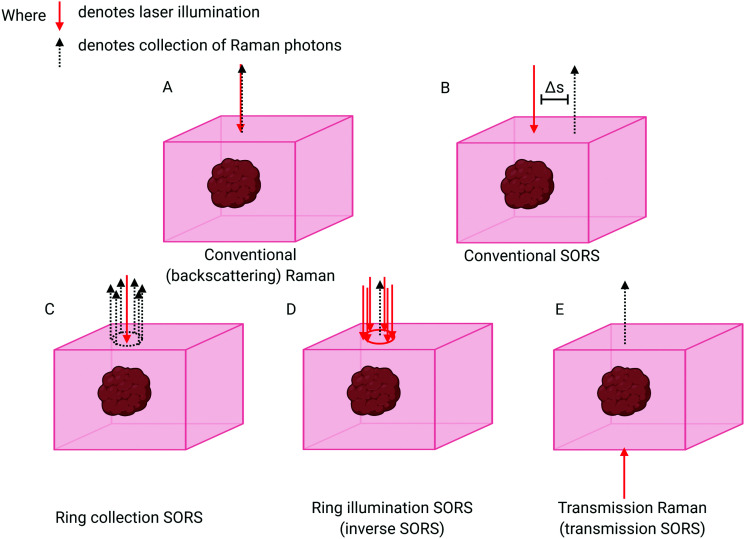
Schematic comparing different SORS approaches with conventional Raman. (A) Conventional backscattering Raman: laser excitation and Raman photon collection take place at the same point. (B) Conventional SORS: the point of collection is offset from the point of excitation by a spatial offset of Δ*s*. (C) Ring collection SORS. The Raman photons are collected in a circular array around the point of excitation. (D) Ring illumination SORS: the laser illumination zone is in the shape of a ring and Raman photons are collected at the centre of the ring. (E) Transmission Raman: the laser excitation and the Raman collection zone are separated to the extreme. Raman collection takes place on the opposite side to laser illumination.

The breakthrough in deep probing of tissue with Raman spectroscopy emerged with the advent of SORS.^5,15,16^ The approach relies on the fact that deeper penetrating photons statistically tend to migrate laterally from the illumination zone on the sample surface, in a random walk-like fashion,^17^ whereas those photons that have scattered back to the surface from shallower depths have had less opportunity to travel laterally. The typical presence of surface-to-air interfaces accentuates this effect by preferentially facilitating loss of the photons propagating through near-surface layers, as any laser photon reaching the interface is immediately and irretrievably lost. Collecting Raman photons at the surface of the sample away from the laser illumination zone therefore biases the detected signal towards deeper zones within the sample. This is in contrast with conventional Raman collection geometry, *i.e.* back-scattered geometry, where signal would be collected from the illumination zone itself (Fig. 1A). The separation between the illumination and collection zones is termed spatial offset Δ*s* (Fig. 1B) and, the larger the spatial offset, the greater the signal bias towards the deeper zones.

If the sample is stratified with chemically distinct layers one can use varying spatial offsets to obtain SORS spectra of individual layers in a sample, thus enabling the chemical composition of each separate layer to be elucidated. This is accomplished by a scaled subtraction of SORS spectra from each other to cancel the contribution of individual layers^6^ and can be carried out in an automated manner without any knowledge of the composition of each layer. Another means of accomplishing this is to subject the set of SORS spectra obtained at different spatial offsets to multivariate analysis. This can facilitate deconvolution of different layers in a semi-automated way, *e.g.* using Principal Component Analysis (PCA) in combination with Band Target Entropy Minimisation (BTEM)^18–22^ to retrieve real spectra of individual layers rather than their linear combinations that are typically output from a basic PCA. Recently a more advanced variant has been developed for specifically retrieving Raman signals from biological samples (bone in soft tissue) and is termed “adaptive BTEM” (ABTEM).^23^ Alternatively, the spectral unmixing can be performed using an overconstrained extraction algorithm based on fitting with spectral libraries.^24^ General applications of SORS in biological areas include the identification of particular biomarkers, or the quantification of some biological subcomponents relative to some other measurable Raman entity in the sample.

The basic SORS concept relies on a point-like illumination of the sample surface and collection of Raman signal from a point-like zone on the sample surface. SORS collection efficiency can be improved by integrating the signal at the same offset from the illumination point, *i.e.* in a circle (Fig. 1C). A more advanced variant of SORS, which has a particular relevance to biomedical applications is ring illumination SORS (Fig. 1D).^25,26^ In this arrangement, the laser illumination zone is in the shape of a ring and Raman signal is collected from the centre of this ring, *e.g.* using optical fibres. The radius of the illumination ring facilitates the spatial offset. A ring shape profile of the laser illumination beam is typically generated using a conical lens (‘axicon’)^26^ and the radius of the illumination zone can be varied, for example, by changing the axicon-to-sample distance. Further, using an axicon in this way leads to varying ring diameters, but an invariant ring thickness, being dependent on the beam diameter incident on the axicon lens. As the larger ring radius brings with it a larger illumination area, this concept enables the increase of the illumination laser power for larger spatial offsets to benefit *in vivo* applications whereby intensity is typically restricted by permitted laser safety thresholds. Such an increase of laser power for larger spatial offsets is highly desired as the larger spatial offset is inherently accompanied by lower overall detectable SORS signals. This benefits accuracy of measurement and enhances penetration depths. Recently, the use of light modulation in SORS imaging facilitated by micro–electro–mechanical systems (MEMS) was also demonstrated. The concept permits rapid switching between different spatial offsets without using moving parts of the system apart from the MEMS subcomponents themselves.^27,28^

When planning SORS measurements one must evaluate the optimum spatial offsets, taking into account the geometry and optical properties of the sample. There are several approaches to finding the optimum offset. In general, the accessible depth typically increases monotonically with increasing spatial offset. However, the larger the spatial offset, the weaker the Raman signal typically becomes. Therefore, a balance must be struck between the two processes to achieve optimum conditions in order to probe tissues at a specific depth. The goal is to achieve the highest signal-to-noise (S/N) ratio in the signal retrieved from a particular (target) layer or depth. This is often accomplished empirically by trial and error and by changing various experimental parameters, *e.g.* laser power or acquisition time. Alternatively, one can perform numerical analysis (*e.g.* photon propagation simulations) to deduce the expected S/N for a particular spatial offset. The latter requires the knowledge of sample optical properties and as such, is a less common approach compared to the former. The setting of optimum spatial offset was studied by Maher and Berger^29^ and Bloomfield *et al.*^30^ The latter study also concluded that for optimum results, a larger acquisition time should be devoted to acquiring SORS spectra at larger spatial offsets in order to obtain the highest possible S/N spectra from a target layer or depth.^30^

With regards to collection of scattered photons, the Raman signal is often collected from the sample surface using optical fibres to decouple the sample from the core detection apparatus, however free space coupling using collection and imaging lenses can also be used. The detection is typically facilitated using a low f-number (high etendue) spectrometer equipped with a deep depletion CCD (maximising quantum efficiency for near-infrared (NIR) light) on its output port.^31^

SORS limitations include restricted penetration depth given by the experimental conditions and sample specific conditions; principal SORS geometry and optical properties of individual layers. In particular, absorption can severely limit accessible depths and is therefore a particular consideration in medical applications. For this reason, SORS measurements are typically performed using a NIR laser excitation (*e.g.* 785, 808 or 830 nm), within the NIR optical window for tissue, to ensure minimum laser and Raman photon absorption.^6,32,33^ Other restrictions on SORS use includes interference from sample fluorescence which can be particularly severe with darker tissues (*e.g.* with liver or higher melanin pigmentation in skin) or due to the presence of hair on the sample (especially if it is of a darker colour). The latter, however, can be mitigated by shaving the probed area.^34^ The fluorescence generated due to the pigmentation associated with darker skin complexions can also be reduced by introducing spatial offsets since it is confined to surface layers where melanin resides when probing deeper layers. In analogy with the relative reduction in the Raman signal from the surface when using SORS, the surface fluorescence signal is also suppressed relative to subsurface signal from the same layer by introducing spatial offsets.^35,36^ Alternative approaches include time gated fluorescence suppression.^37–40^

### Micro-SORS

The use of micro-SORS which combines SORS with microscopy to probe very thin layers on a micrometre to sub-mm scale has also been demonstrated. The technique has been applied extensively to the analysis of paint in cultural heritage and also exemplified in analysis of wheat seeds and in other areas.^41–44^ The technique of micro-SORS is mentioned here for completeness as it is reasonable to assume that studies involving humans would require the probing through larger thicknesses than what can be achieved using micro-SORS. However, micro-SORS could be beneficial for probing very thin interfaces such as skin in dermatology at depths beyond the reach of conventional confocal Raman microscopy, or with smaller animal models. This could also be advanced through the development of “flexible” Raman instrumentation to enable sampling of areas of the body which are otherwise difficult to reach.^45^

### Transmission Raman

“Transmission Raman Spectroscopy” (TRS),^46^ in which the laser excitation area and Raman collection area are located on opposing sides of the sample, can also be considered an extreme case of SORS (Fig. 1E). The technique can only be applied when access to the two sides of sample is available and, importantly, where sample thickness permits Raman signals of adequate quality to be acquired. Unlike SORS, in its simplest form, TRS does not permit the separation of sample layers from each other. It instead enables an approximation of a volumetric Raman signal to be obtained. Although TRS was demonstrated in very early days of Raman spectroscopy its utility in the medical field has only recently been recognised and exploited.^6,47,48^

### Surface enhanced spatially offset Raman spectroscopy (SESORS)

Another biomedically relevant variant of SORS is “surface-enhanced spatially offset Raman spectroscopy” (SESORS), a technique which combines the depth penetration capabilities of SORS with the signal enhancing benefits of SERS to generate Raman signals through depths far greater than what can be achieved using traditional SERS imaging. SESORS has previously been shown to access depths of ∼5 cm in biological tissues, thus providing exceptional depth sensitivity and selectivity.^49,50^

**Fig. 2:**
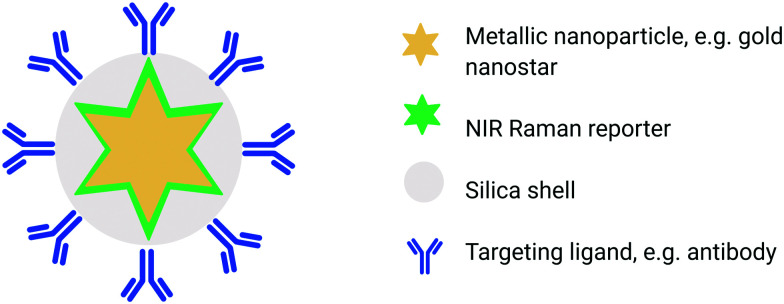
Conceptual figure outlining the design of SE(R)RS NPs for *in vivo* applications. SE(R)RS NPs typically consist of a gold core, *e.g.* star which is then functionalised with a Raman reporter molecule and encapsulated in a thin silica shell. Active targeting is achieved through the use of a specific targeting ligands such as antibodies.

In order to carry out SESORS measurements, SERS-active nanoparticles (NP's) must first be synthesized from a metallic NP, typically gold, which is then functionalized with a Raman reporter molecule.^51^ SERS relies on the interaction between the analyte (*e.g.* Raman reporter molecule) and the localised surface plasmon resonance (LSPR) asscoiated with the metallic NP onto which the analyte is adsorbed. Following interaction with incident light, oscillation of the conduction band electrons takes place which increases the local field experienced by the NP. In turn, this induces greater polarisation of the analyte molecule and thus further enhancement in Raman scattering consequently observed.^52^ Moreover, by functionalizing the metallic NP with a reporter molecule that is in resonance with the wavelength of incident light, it is possible to achieve enhancements in signal by even greater orders of magnitude (10^10^–10^14^).^51,53–55^ This is referred to as “surface enhanced resonance Raman scattering” (SERRS). Although intravenous administration of the SE(R)RS NPs into a living subject is an invasive procedure, imaging of the accumulation of SE(R)RS NPs at the site of interest by means of SORS is non-invasive and can also be carried out repeatedly.^54^ In addition, SESORS measurements have also been performed through the use of a metallic micro-substrates implanted subcutaneously to enable repeated measuring of blood glucose levels.^56^ It is also possible to engineer SE(R)RS NPs to specifically accumulate at the site of interest *in vivo* through the use of targeting ligands such as antibodies^57–60^ or peptides.^61^ In this instance the SE(R)RS NPs are typically encapsulated in a silica shell prior to biomolecule functionalization, (Fig. 2).^55^ This is of interest in diseases such as cancer in which SE(R)RS NPs have been shown to specifically target biomarkers that are over-expressed on the surface of tumour cells.^34,61,62^ As such, SESORS is a promising technique which can offer, deep, molecularly specific, non-invasive *in vivo* imaging.

### Special techniques (temperature, pH and depth)

Various other derivatives of the above methods have also been introduced in recent years which enable the monitoring of other sample parameters rather than just chemical composition. These include temperature (*T*) and pH sensing as well as depth determination of an inclusion within a turbid matrix.

For example, the monitoring of temperature can be achieved by utilizing a combination of Stokes and anti-Stokes measurements to detect changes in the temperature of a target (*e.g.* SE(R)RS NPs). Such an approach has been demonstrated with single SERS NPs,^63^ and more recently through greater depths using SESORS with both reporter NPs and photothermal gold nanoshells.^64–66^

In some applications it is also desirable to localise the target (*e.g.* lesion) in a matrix (*e.g.* tissue). This can be achieved, in part, by moving the sample in the *x* and *y* directions and maximising SORS or TRS signals. However, determining the specific depth of a target in the *z*-direction is more challenging. Recently, a simple yet effective concept relying on differing absorption of two (or more) Raman bands of the lesion by the surrounding matrix was demonstrated and can be applied to samples with or without SERS NPs.^65^ The deeper the phantom lesion is located within the tissue, the longer the propagation distance of emerging Raman photons at tissue surfaces (points of collection). Moreover, longer propagation distances result in larger relative Raman band intensity distortion of the target signal due to tissue differential absorption.^67,68^ This is a result of water and/or lipids absorbing the two monitored Raman bands differently. The application of these special techniques for the monitoring and assessment of depth, pH and temperature are discussed in the “Applications” section below.

## Applications

Although the SORS technique is currently not approved for general diagnostic use in patients, significant advancements in this area have been made with the aim of eventually translating this technique into the clinic. In this section, we will discuss recent examples in which SORS has been applied to solving medically related problems within the last five years.

### Assessment of blood quality

In combination with SERS substrates, one of the first practical applications of the SESORS technique was for the non-invasive monitoring of blood glucose levels *in vivo*.^56,69^ Recent advances have also shown that SORS can be applied to other blood-related questions, specifically the quality assessment of red blood cells for patient transfusion. Typically, following donation and separation into different components, red blood cell concentrates can be stored for up to 42 days before they must be discarded due to concerns over biochemical changes.^70,71^ By utilizing SORS, Vardaki *et al.*, demonstrated that it is possible to profile changes in oxygenation over the 42-day storage period. It is well understood that over time, the chemical composition of blood units from each donor changes at different rates, thus the age of the unit does not provide certainty with regards to red blood cell quality.^71^ Importantly, SORS analysis provided a means to non-invasively monitor and provide useful chemical information on the quality of blood at a particular time, *e.g.* prior to transfusion, without compromising the usability of the unit. These results demonstrate the suitability of SORS to facilitate quality-control in blood-banks.

### Assessment of bone diseases

Since the first demonstration of SORS in 2005, one area that has seen intense investigation in recent years is the use of SORS for the non-invasive assessment of bone disorders such as osteoporosis.^25,35^ Currently, the clinical standard for measuring bone density is by dual X-ray absorptiometry (DXA) which measures areas of low bone mass and thus identifies patients with increased fracture risks.^72,73^ However, such an approach has been shown to be a poor indicator of low bone mass in certain circumstances, *e.g.* early postmenopausal women,^72,74^ and in addition, requires the use of ionizing radiation. Moreover, DXA is unable to measure the organic component of bone, mainly collagen, meaning, at least in part, that around 30–40% of fracture risks go undetected.^25^ Quantitative CT can be used as an alternative since it is able to provide volumetric and stress–strain information, however in comparison to DXA, it is associated with significantly higher levels of radiation and greater financial costs.^72^ Thus, due to its lack of ionizing radiation, low cost and ability to provide information on both the inorganic and organic component of bone (mineral and collagen respectively), SORS has been explored as a promising alternative.

A SORS tomographic instrument was designed in which excitation and collection fibers were assembled in a 360-degree plane around the sample of interest. In combination with microCT, this arrangement of fibers enabled the transcutaneous tomographic Raman imaging of intact rat tibia.^75^ As well as the use of microCT,^75^ other reports have also highlighted the benefit of combining SORS with other imaging modalities such as optical coherence tomography (OCT),^76^ to help support the obtainment of morphological and molecular information from deeper layers of biological samples. Feng *et al.*, applied SORS to measure subcortical bone tissue and biochemical changes through increased depth using intact murine bones.^77^ Importantly, the same approach was later applied to predict bone strength transcutaneously, *in vivo*.^78^ Using partial least squares (PLS), it was possible to predict area mineral bone density, volumetric bone mineralization density and maximum torque of each tibia as quantified *ex vivo* using DXA, microCT and biomechanical tests respectively. Although several reports have successfully demonstrated the potential of SORS to identify changes in both the organic and inorganic component of bone, it is extremely important to understand the precise depth at which particular Raman photons are generated in order to correctly assign any observable changes to the surface or sub-surface sample components. This is vital if SORS imaging is to translate into the clinic. Sowoidnich and co-workers performed studies to evaluate the sampling depth of a SORS set up with bone samples using segmented bones and inserting a thin interlayer at different depths.^79–81^ Investigation into the influence of bone mineralization on photon migration properties from *ex vivo* bone samples with varying levels of mineral density revealed that photons can more easily migrate inside less mineralized bone. The maximum accessible sampling depth was also found to be dependent on the bone mineralization levels, therefore in comparison to the healthy controls, Raman signals could be detected through greater thicknesses of less mineralized, unhealthy bone.^79^

Buckley *et al.*, reported the potential of SORS to detect differences in the mineral component of bone in fractured and non-fractured controls. The results demonstrated that fractured femora were 5–10% more mineralized than non-fractured controls.^82^ The ‘mineralisation term’ refers here to the ratio of mineral to collagen which was measured to be higher for fractured bones. This is in line with expectations, as less minerals compared to collagen makes the bone matrix more brittle. Recently, the use of adaptive-band targeted entropy mineralization has been applied to spectral un-mixing of SORS spectra from bone samples buried beneath tissue.^23^ The algorithm was successfully applied to transcutaneous SORS spectra and represents a step towards development of an optimized clinical SORS system, specific for diagnosis of bone disease in patients.

**Fig. 3:**
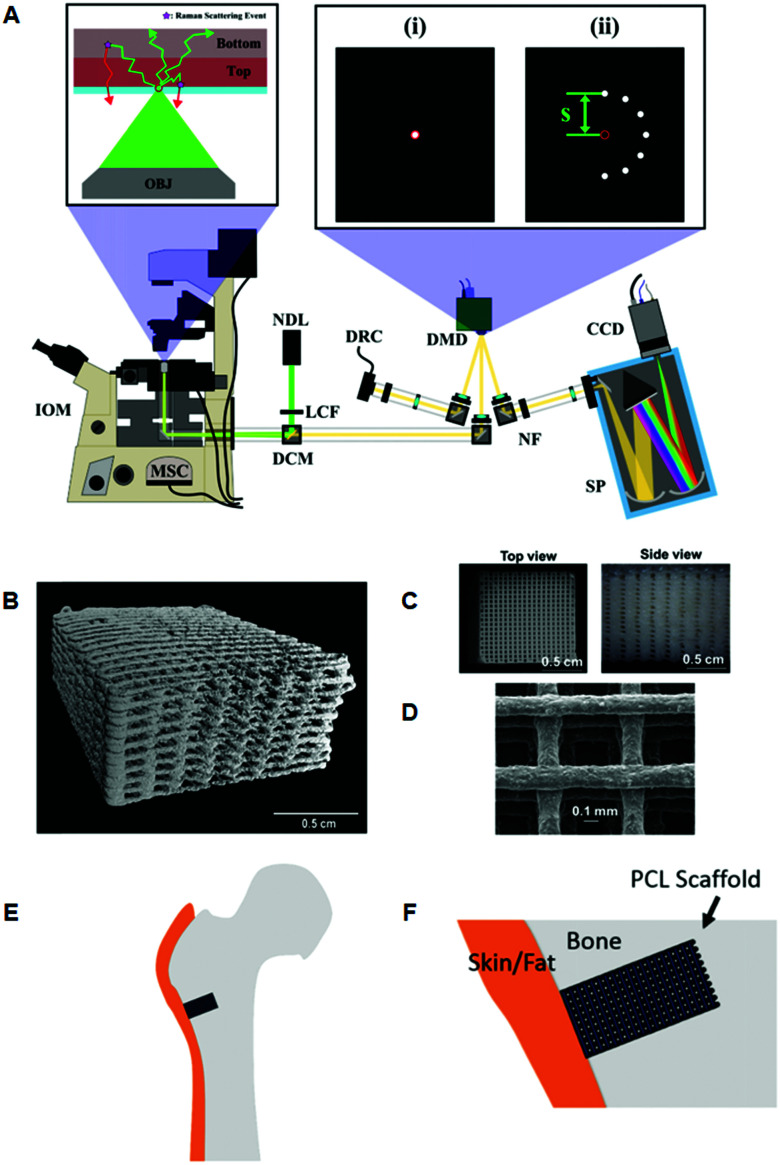
Representative images of 3D printed bone scaffolds. (A) Graphical drawing of the DMD-based SORS instrument used to image the bone scaffolds. Abbreviations are as follows; OBJ, objective; IOM, inverted optical microscope; MSC, microscope side-port camera; DCM, dichroic mirror; LCF, laser clean filter; NDL, 20 mW, 532 nm Nd:YAG diode laser; DMD, digital micro-mirror device; DRC, DMD inspection camera; NF, notch filter; SP, spectrometer; CCD, charge couple device detector. The red circles in the magnified images (i) and (ii) refer to the position corresponding to the focused laser on the sample, and on the sample-conjugate plane of the DMD (equivalent to a zero spatial offset). (i) Displays a DMD pattern for a standard confocal Raman measurement and (ii) shows a possible SORS configuration. (B) Micro-CT 3D reconstruction image of polycaprolactone (PCL) and hydroxyapatite (HA) scaffold PCL : HA 1 : 4 with dimensions 10 × 10 × 5 mm, porosity = 64.7%. (C) Images from dissecting microscope of a PCL : HA (1 : 4) scaffold (D) scanning electron microscopy of a PCL : HA scaffold with 1 : 2 blend ratio. (E) Conceptual diagram of the head of a femur with a critical defect drilled into it. The scaffold is then inserted into the defect. (F) Conceptual close up of the bone defect filled with a PCL scaffold. Adapted from ref. 83 and 84 with permission from The Optical Society, copyright 2016 and 2019.

Using a programmable digital micro-mirror device (Fig. 3A) to collect or reject light at spatial positions in a 2D plane, Liao *et al.*, developed a fast method of setting spatial offsets in SORS measurements of diverse shapes.^83^ This was then applied to the non-destructive characterization of bone tissue engineering scaffolds, (Fig. 3B–F).^27^ Optimization was achieved using a series of phantoms to mimic the mineralization of scaffolds implanted in a critical bone defect of a large animal, and to further understand the measurement conditions required for an *in vivo* longitudinal study. Such a concept represents a promising approach towards the longitudinal monitoring of engineered bone scaffold mineralization and bone re-growth *in vivo*.^84,85^

### Monitoring of changes in temperature and pH

The determination of temperature is particularly relevant to photothermal therapy (PTT) applications, where currently no practical method exists for reading subsurface temperatures of SERS NPs mediating PTT and surrounding tissue.^86^ To date, PTT is performed without any real time feedback on temperature changes taking place through significant (>few mm) depth. Moreover, temperature is highly dependent on sample geometry and the optical properties of both the medium, *e.g.* tissue and the photothermal material, *e.g.* NPs, thus making it difficult to control. However, since SORS accounts for sample geometry and optical properties, the combination of Stokes with anti-Stokes measurements can therefore enable monitoring of temperature changes in an analyte (*e.g.* SERS NPs).^64,66^ Surrounding bulk tissue temperature can also be monitored by measuring directly the Stokes and anti-Stokes bands or those of SERS NPs labelled or assembled in such a way so that they do not absorb and mediate the heat transfer from the laser heating source. A proof-of-concept study has also demonstrated that it is possible to monitor pH deep within tissue by monitoring the position of a SESORS band of NP's labelled with molecules exhibiting Raman band position pH-dependency.^87^ More recently, a similar approach showed that it was possible to also precisely predict the depth at which SERS NPs were buried in a turbid phantom (0.5% intralipid) and simultaneously monitor changes in pH of the media surrounding the NPs.^88^

### Detection of neurotransmitters

**Fig. 4:**
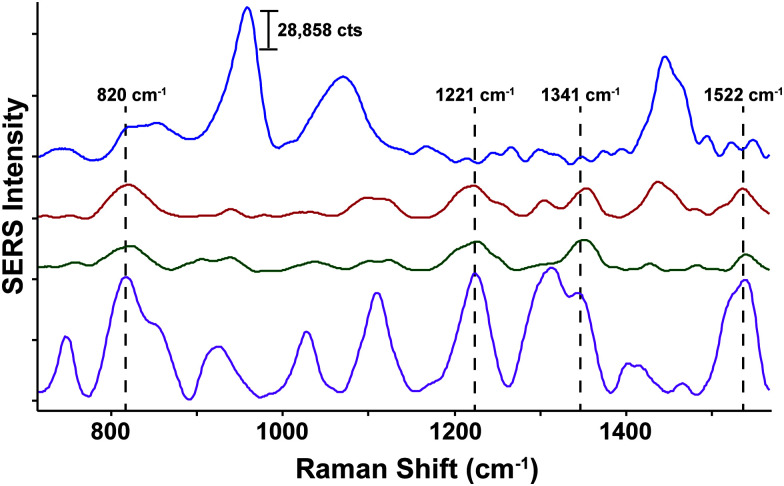
*Ex vivo* detection of 100 μM serotonin through the skull of a mouse using SESORS. A SORS spectrum of the mouse brain through the intact skull without the injection of serotonin or gold NPs (top spectrum, blue). A SESORS spectrum of the mouse brain following the injection of 100 μM serotonin at a 0 mm offset (red) and a 1 mm offset (green). The SORS spectrum of the mouse brain before the injection of NPs was subtracted from the offset spectra. Data was acquired using a laser wavelength of 785 nm, 90 mW, *t* = 120 s. The SERS spectrum of 100 μM serotonin on AuNPs at pH 2, 785 nm, 5 mW, *t* = 30 s (purple). Reproduced from ref. 92 with permission from the Royal Society of Chemistry, copyright 2020.

Neurotransmitters are a class of chemical messengers which transmit signals across a chemical synapse from one neuron to another target neuron, muscle cell or gland cell.^89^ They play a critical role in maintaining human health and any imbalance can cause serious mental or physical health conditions including schizophrenia and Parkinson's disease. Currently, there is a need to establish more rapid, effective methods capable of quantifying neurotransmitter concentrations *in vivo*. With this in mind, SESORS has since been utilized for the measurement of melatonin, serotonin and epinephrine at concentrations as low as 100 μM in a brain tissue mimic through a cat skull.^90^ Using an inverse SORS approach, the *ex vivo* detection of SERS NP through a monkey skull has also been reported.^91^ More recently, Sharma and colleagues reported the ability to resolve spectral signatures from individual neurotransmitters and mixtures of neurotransmitters at physiologically relevant concentrations using inverse (ring illumination) SORS. This was achieved using agarose gel which was then embedded within the *ex vivo* rat skull to create a brain tissue mimic. Using these phantoms, SESORS established limits of detection for a range of neurotransmitters at physically relevant concentrations; melatonin (100 nM); serotonin (400 nM); dopamine (1 μM); norepinephrine (400 nM); and epinephrine (800 nM).^92^ In addition, the brain of a sacrificed mouse was spiked with serotonin and gold NPs to demonstrate the potential of SESORS to detect neurotransmitters through the skull, (Fig. 4).^92^ This represents an notable step towards the use of SESORS for the *in vivo* detection and continual monitoring of varying concentrations of neurotransmitters in a living system. However, it is important to note the challenges associated with the detection of low concentration species directly using SESORS *in vivo*. This is in part, due to complex nature of biological fluids in which several components, *e.g.* proteins, will compete with the neurotransmitters in order to bind to the gold NP surface.

### Cancer diagnostics using SORS

Owing to its ability to provide chemically specific information in a non-invasive manner, previous work has demonstrated the applicability of SORS and transmission Raman for the non-invasive detection and characterization of cancerous lesions.^93^ With the exception of skin cancer, breast cancer is the second most common cancer to affect women the United States^94,95^ and screening is typically performed using mammography (low energy X-rays) to locate tumors inside the breast.^96^ MRI may also be used to screen women who are associated with a higher risk of breast cancer however both imaging techniques are associated with false positives. In addition, they also fail to provide molecularly specific information meaning that invasive biopsies must be performed in order to confirm the disease status which results in increased patient anxiety and costs.

**Fig. 5:**
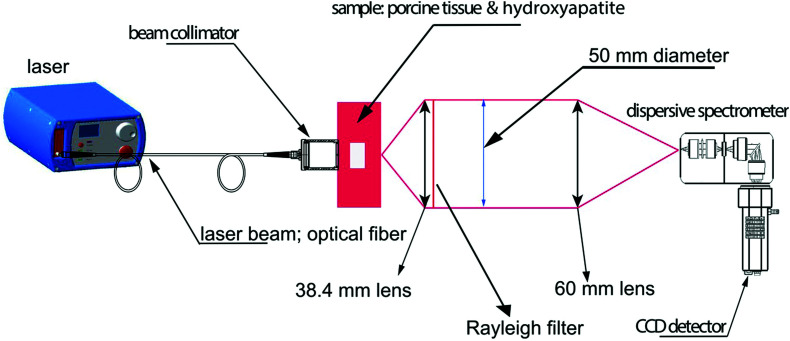
Conceptual figure describing the transmission Raman experimental set-up. Reproduced from ref. 31 with permission from John Wiley & Sons, copyright 2018.

Two major types of microcalcifications are found in breast tissue: calcium oxalate dihydrate (type 1) and calcium phosphates, mainly calcium hydroxyapatite (type 2) with the latter resulting from cellular degradation or necrosis and is thus associated with a higher likelihood of malignancies.^97^ Raman spectroscopy has been shown to chemically identify differences in benign and malignant microcalcifications of the breast and SORS and transmission Raman has since been applied to identify such calcifications at clinically relevant depths.^93,98,99^ Previous research into this application reported the possibility to detect hydroxyapatite at clinically relevant concentrations through a depth of 20 mm,^98^ however more recently, optimization of instrumentation (Fig. 5) enabled detection of clinically relevant concentrations of hydroxyapatite through a depth of 40 mm.^31^ This was achieved by increasing the power of the incident laser through use of a larger spot size, improving the efficiency of the spectrograph by increasing the slit width and utilizing a grating with a high dispersion to improve spectral resolution. These results have helped drive the translation of this technique into the clinical settings with this approach now moving towards trials in humans.^100^

**Fig. 6:**
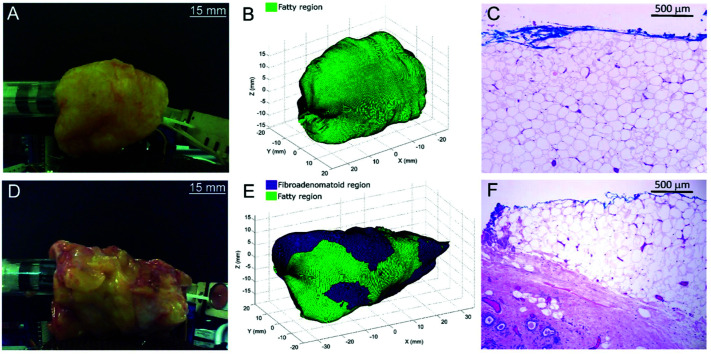
3D margin assessment of breast specimens using automated depth-averaged Raman spectroscopy. (A) Photograph of the breast specimen which includes fatty margins. (B) Margins of the specimen as rendered by the 3D-scanner. (C) H&E section from a biopsy spot taken from the fatty margin (10× magnification). (D–F) Describe the corresponding figures for a breast specimen with fibroadenomatoid margins. (F) An increase in fibro-epithelial composition can be observed at a depth of 0.5–1.5 mm from the margin. Reproduced from ref. 101 with permission from Nature Research, copyright 2017.

Thomas *et al.*, evaluated the feasibility of a 3-dimensional (3D) scanner to assess the entire margins of a resected specimen within a clinically feasible time.^101^ Building on a previous design by Keller *et al.*,^102^ the authors designed a 7 mm diameter probe that enclosed 36 detector fibres (100 micron in diameter) organised into 4 quadrants.^101^ The 36 fibres were then aligned into a single line at the spectrograph input. In comparison to the previous probe design which contained only 10 fibres,^102^ the new design contained 36 optical fibres and therefore facilitated approximately four times faster collection of Raman photons with the same S/N ratios. Furthermore, in comparison to previous work, it was possible to acquire Raman signal from a larger surface area of 38.5 mm^2^, thus enabling the probe to cover the entire specimen surface over a shorter period of time. Using breast tissues excised from prophylactic mastectomy specimens and a classical least squares algorithm, areas of the specimen were assessed and margins were classified either as fatty or fibroadenomatoid (Fig. 6A, B, D, and E). 3D images of the sample obtained using depth-averaged Raman imaging were also in agreement with histopathological staining, (Fig. 6C and F). The results demonstrate the suitability of a SORS-based probe to carry out depth-averaged Raman imaging for the assessment of biochemical margins of disease.

### Cancer diagnostics using SESORS

SORS provides a means of detecting the chemical signatures of diseased tissue through depth in a non-invasive manner. Moreover, it is widely accepted that if the intended application is to detect Raman signals at the site of interest through depth, SORS offers a clear advantage over traditional Raman imaging approaches.^103,104^ In addition to SORS and in combination with SERS NPs, SESORS imaging has also been explored for the detection of disease through tissues. In this instance, however, it is not the spectral signatures of the diseased tissue that are detected, but instead the molecularly specific “fingerprint” spectra of the Raman reporter molecules functionalized to the surface of metallic NPs, namely gold.

**Fig. 7:**
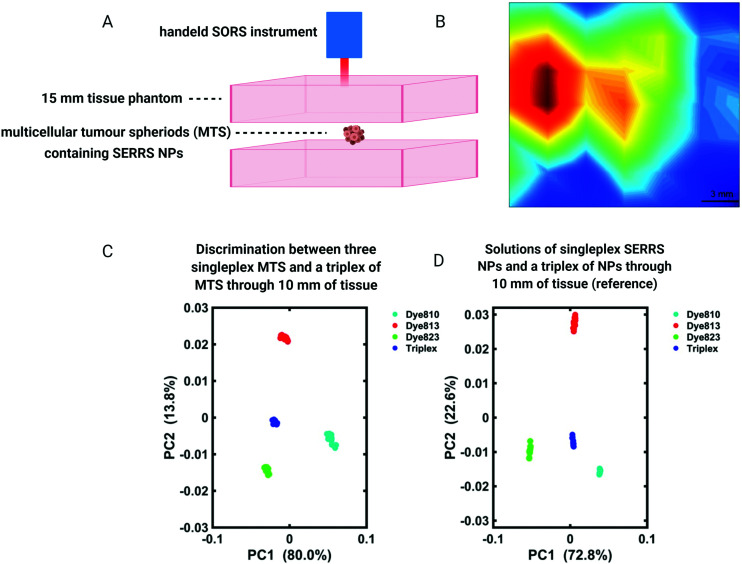
(A and B) A false colour *xy*-2D heat SESORRS map of MTS containing SERRS NPs through 15 mm of tissue using a handheld spectrometer. Mapping was performed using a 3 mm step size to create an image of 7 × 7 pixels. (C and D) PC scores plots discriminating between single SERRS NP flavours and a triplex of all three SERRS flavours within (C) MTS and (D) solution through 10 mm of porcine tissue. Distinct separation is observed in both score plots. All measurements were carried out using a 2 s integration time, 5 accumulations, 830 nm laser excitation wavelength. Adapted from ref. 105 and 106 with permission from the Royal Society of Chemistry, copyright 2018.

For the detection of diseases such as cancer, SESORS relies on the accumulation of SERS NPs at the site of interest, *e.g.* tumor. Using *ex vivo* MCF7 multicellular tumor spheroids (MTS) as 3D tumor models of breast cancer, the detection of SERRS NPs which had accumulated inside MTS through depths of 15 mm was demonstrated (Fig. 7A and B).^105^ In this instance MTS were buried beneath porcine tissue phantoms and mapped using a handheld SORS spectrometer to demonstrate the potential suitability of handheld SORS instrumentation for use in the clinic. The advantage of using resonant chalcogenpyrylium-based Raman reporters to generate high Raman scattering through depth was also shown, enabling the authors to report the application of “surface-enhanced spatially offset resonance Raman spectroscopy” (SESORRS) for the first time.^105^ Using the same set-up, the ability to spectrally distinguish between multiple SERRS NPs within MTS was also explored.^106^ Through the application of PCA it was possible to not only detect but classify multiple vibrational fingerprints through a depth of 10 mm (Fig. 7C and D). Reference spectra were obtained from aqueous solutions of SERRS NPs containing each of the three single SERRS NP flavours and a mixture composed of equal amounts of each solution (triplex), obscured behind 10 mm of porcine tissue. Moreover the benefit of using resonant Raman reporters for SESORRS has also been shown to improve limits of detection.^107^ In comparison to the non-resonant Raman reporter molecule 1,2-bis(4-pyridyl)ethylene (BPE), NPs functionalized with resonant chalcogenpyrylium reporter molecules can be detected at much lower concentrations using SESORRS. In this report, calculated limits of detection indicated that such SERRS nanotags stored in a glass cuvette and obscured by 5 mm of tissue could be detected at concentrations as low as 104 fM using SESORRS.^107^

**Fig. 8:**
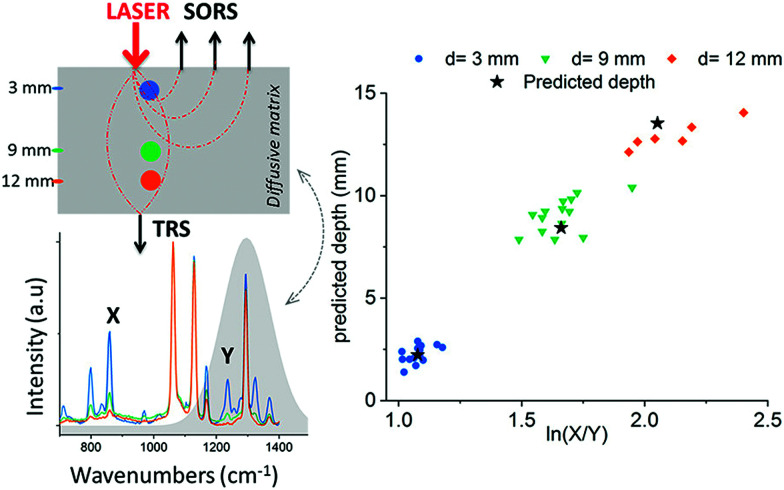
Prediction of paracetamol depth in a turbid matrix using conventional SORS and transmission Raman measurements. Results show conventional SORS and transmission Raman predicted *vs.* measured depth of paracetamol buried at depths of 3, 9 and 12 mm. Measurements were acquired using 20 s integration time, 5 accumulations and a laser output power of 200 mW (830 nm). Reproduced from ref. 67 with permission from the American Chemical Society, copyright 2019.

In these instances, however, the SERRS NPs were buried at specific depths, meaning that the Raman signals acquired from the SERRS NPs were detected from known and predetermined depths. To date, the ability to precisely determine the specific depth at which SERRS NPs are located without any prior knowledge remains a significant challenge and the means to do so is especially important for *in vivo* applications where SERRS NPs will be distributed at different and unknown depths, *e.g.* within a cancerous lesion.^108^ Using SORS and transmission Raman, Mosca *et al*, recently reported a viable and robust method capable of predicting the depth of both a single buried object and SERRS NPs through turbid phantoms by monitoring the relative intensity of two Raman bands exhibiting differential absorption by the matrix (Fig. 8).^67,68^ A proof-of-concept study demonstrated possibility of achieving better than ∼10% accuracy of determining the depth of a buried target relative to the overall sample thickness in SORS and TRS measurements.^67^ The results demonstrate a potential effective means of calculating the depth at which SERRS NPs are localized *in vivo* as well as determining the optimum distribution of laser radiation around a sample in PTT applications.^68^

**Fig. 9:**
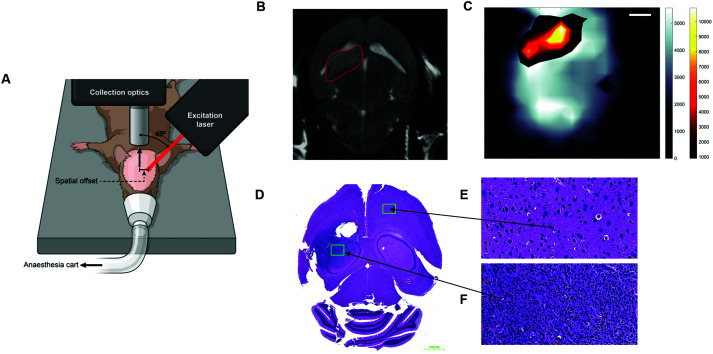
*In vivo* SESORRS imaging of glioblastoma multiforme through the intact skull of mouse. (A) Conceptual figure demonstrating the SORS set-up. (B) 2D axial T2-weighted MRI taken 4 weeks post injection of DF-1 cells which induce the growth of GBM *in vivo*. MRI confirms the presence of a left frontal tumour (outlined in red). MR Images were acquired using a slice thickness of 0.7 mm taken at a depth of 3.6 mm. (C) The SORS heatmap of the SERRS NPs was superimposed onto the SORS heat map of bone with the SESORRS image delineating the tumour margin in agreement with the MR image. (D) H&E stained 5 μM section of the brain. The arrows correspond to areas of the slice which represent healthy tissue (E) and unhealthy tissue (F). Images in (E and F) were taken at 40× magnification. SORS measurements were acquired by utilizing a power density of 13.8 mW mm^−2^ (785 nm), a spatial offset of 2.5 mm, 3 s integration time, 5 acquisitions. Reproduced from ref. 34 with permission from Ivyspring International Publisher, copyright 2019.

Several reports in the literature have also discussed the potential of SESO(R)RS to image disease, specifically cancer, however, until recently, this approach was only applied to *ex vivo* phantoms and not to a living system, *i.e. in vivo*. To address this, Nicolson *et al.*, demonstrated the ability to image glioblastoma multiforme (GBM) in living mice, non-invasively, using the SESORRS technique (Fig. 9A).^34^ Using a transgenic mouse model, which presents with all the histopathological and imaging hallmarks of human high-grade gliomas, the authors used cyclic RGD peptides to actively target and thus image GBM *in vivo* through the intact skull. A classical least squares fit was used to support image analysis and generate SESORRS images that outlined the tumors with high precision. SESORRS images (Fig. 9C) were in agreement with magnetic resonance images (Fig. 9B) taken prior to the injection of SERRS NPs, as well as *ex vivo* histology (Fig. 9D–F).^34^ Not only is this work the first demonstration of SESORRS for the *in vivo* imaging of any disease, it also represents an important step towards the clinical translation of SE(R)RS NPs by demonstrating the ability to detect them *in vivo* through depths far superior to traditional Raman imaging methods. Using subcutaneous models, previous work has demonstrated the use of traditional Raman mapping to detect both single and multiple flavours of SE(R)RS NPs simultaneously *in vivo*.^54,57,58,109,110^ In these instances, however, the tumours were located at shallower depths. We therefore envisage that due to the ability of SESO(R)RS to probe through greater depths and thus detect deeper-seated tumours, we will see an increasing number of research groups utilizing this technique for a wide range of pre-clinical imaging applications. Specifically with regards to cancer imaging, we encourage the development of brighter SE(R)RS NPs as well as strategies which will enable greater NP accumulation within the tumour microenvironment. This can be achieved by recognizing tumours as complex and heterogeneous structures, rather than simply as a leaky sponge in which NPs will accumulate following prolonged circulation *in vivo*. Consideration of the above, as well as a number of other factors, will also help to reduce off-target accumulation and potential long-term toxicity *in vivo*.^111^

## Conclusion and outlook

In recent years, the suitability of conventional SORS and transmission Raman for the non-invasive analysis of disease has advanced to an extremely exciting level. In this review we have discussed the use of SORS for the detection of bone disease, neurotransmitters and cancer, however, in the future, we envisage that the application of SORS and SESO(R)RS imaging will not remain exclusive to these three areas. We are particularly excited by the prospect of using SESO(R)RS as a tool to complement the limitations associated with other clinically approved molecular imaging techniques. Whilst we note the translation of SESORS into the clinical setting is highly dependent on the approval of SE(R)RS NPs by regulatory bodies, such as the Food and Drug Administration (FDA), we believe that advances in SESO(R)RS imaging will help to achieve such approval. Moreover, we anticipate that the field will see a greater shift towards the use of SORS over conventional Raman for applications involving preclinical and clinical imaging. This will be supported through advancements in Raman instrumentation and, in the case of SESO(R)RS, through the development of brighter and safer SER(R)S NPs.

## Conflicts of interest

M. F. K. has several issued and pending patents in the field of SE(R)RS nanoparticles and instrumentation, and is co-founder of RIO Imaging, Inc., a startup company aiming at translating SERRS nanoparticles into the clinic (which did not contribute financially to this work). PM and NS have several issued and pending patents in the field of SORS and related areas.

## Supplementary Material
